# Snakes and Ladders: Unpacking the Personalisation-Privacy Paradox in the Context of AI-Enabled Personalisation in the Physical Retail Environment

**DOI:** 10.1007/s10796-023-10369-7

**Published:** 2023-01-14

**Authors:** Ana Isabel Canhoto, Brendan James Keegan, Maria Ryzhikh

**Affiliations:** 1 University of Sussex, Sussex, UK; 2 Maynooth University, Maynooth, Ireland; 3 Weber-Stephen Products EMEA GmbH, Berlin, Germany

**Keywords:** Artificial intelligence, Personalisation, Privacy, Personalisation-privacy paradox, Retail, Geo-location

## Abstract

Artificial intelligence (AI) is expected to bring to the physical retail environment the kind of mass personalisation that is already common in online commerce, delivering offers that are targeted to each customer, and that adapt to changes in the customer’s context. However, factors related to the in-store environment, the small screen where the offer is delivered, and privacy concerns, create uncertainty regarding how customers might react to highly personalised offers that are delivered to their smartphones while they are in a store. To investigate how customers exposed to this type of AI-enabled, personalised offer, perceive it and respond to it, we use the personalisation-privacy paradox lens. Case study data focused on UK based, female, fashion retail shoppers exposed to such offers reveal that they seek discounts on desired items and improvement of the in-store experience; they resent interruptions and generic offers; express a strong desire for autonomy; and attempt to control access to private information and to improve the recommendations that they receive. Our analysis also exposes contradictions in customers’ expectations of personalisation that requires location tracking. We conclude by drawing an analogy to the popular Snakes and Ladders game, to illustrate the delicate balance between drivers and barriers to acceptance of AI-enabled, highly personalised offers delivered to customers’ smartphones while they are in-store.

## Introduction

Artificial intelligence (AI) is expected to transform business practice in in-store retailing (Davenport et al., 2020), by bringing to the physical retail environment the kind of mass personalisation that is already common in online commerce (Kumar et al., 2017). Personalisation benefits retailers because targeted messages get noticed amid the noise of other communications (Balan & Mathew, 2020), increase sales, and support customer intimacy, involvement with the brand (Gardino et al., 2021) and customer loyalty (Pappas et al., 2018). Moreover, campaign response can be monitored directly and corrective action can be taken promptly, thus improving conversion rate (Chou & Shao, 2021). In the physical retail environment, personalisation is typically provided by the salesperson, which has several limitations. On the supply side, sales staff have access to limited customer data in-store which constrains their ability to adapt their recommendations (van de Sanden et al., 2019). On the demand side, increasingly, customers do not want to interact with a salesperson, particularly in the wake of the Covid-19 pandemic (Mondada et al., 2020; Yoganathan et al., 2021). Where technology is used for in-store recommendations, but not drawing on AI, these are based on customer segmentation rather than individual behaviours. Moreover, such recommendations tend not to reflect real time changes in the context, such as the customer’s location, the store’s inventory levels or the level of crowding in specific area. AI can overcome these limitations of in-store personalisation, due to its ability to integrate multiple sources of information, and create data-driven offers (Kietzmann et al., 2018). Moreover, given that many retail customers use their mobile phones while shopping (Rippé et al., 2017), retailers can deliver the AI-created, targeted messages to customers’ mobile devices while they are in—or near – their store. We refer to this type of targeted offer, which has been personalised by artificial intelligence technology and is delivered to individual shoppers’ phones, in the physical retail environment as “artificial intelligence enabled personalisation” (hereafter referred to as AI-EP).

While there is a rich body of work examining consumer experiences of personalisation in the online environment (see Boerman et al., 2017 for a review), this has not been replicated for physical retail (van de Sanden et al., 2019). However, attitudes towards personalisation vary significantly with the context in which it takes place (Aguirre et al., 2016). First, as consumers’ motivations vary for online vs in-store retail (Haridasan & Fernando, 2018), their perceptions and evaluation of personalisation in the physical environment may differ from those identified in the extant literature on personalisation. Second, the interface through which the message is delivered influences the perception of the extent to which the message has been personalised, with high quality interfaces increasing the perception of personalisation (Ameen et al., 2022). The small screen of mobile phones may impact negatively on consumers’ involvement with the message (Grewal et al., 2016), offsetting their suitability as targeting devices. Third, privacy concerns negatively impact consumers’ evaluation of personalisation in online shopping environments (Li et al., 2017). However, paradoxically, this effect was not detected in Ameen et al (2022)’s study of consumer interactions with smart technologies in shopping malls. In summary, while, from a technical perspective, AI-EP may be similar to online personalisation, factors related to the context of message delivery (in-store), the format of message delivery (small screen) and the salience of privacy concerns in different media suggest that consumer acceptance of personalisation may vary significantly across the two environments. This uncertainty represents a limitation in the current conceptual understanding of consumer acceptance of personalisation and is also a key barrier to adoption AI by businesses (Bughin et al., 2017). That is why Ameen et al (2022), Riegger et al (2021) and van de Sanden et al (2019), among others, have called for empirical research examining consumers’ attitudes towards AI-EP.

This paper aims to advance the conceptual understanding of AI-EP by investigating the following research question: “*How do consumers experience and respond to AI-EP*?”.

To frame this investigation, we draw on the personalisation-privacy paradox, particularly Sutanto et al’s (2013) research on smartphone users. This lens allows us to go beyond understanding whether consumers accept or reject AI-EP, to identify the reasons for their behaviour, as well as how they manage any tensions that may arise while interacting with AI-EP, as urged by Riegger et al (2021). We investigate these dynamics empirically by focusing on a UK fashion retail personalisation app. We focused on one specific app in order to develop an holistic understanding of the usage climate of this technology, as recommended by Wang et al (2015). We chose fashion retail because this is a highly dynamic industry, which benefits from targeted, location-based communication with customers (Kumar et al., 2017); and because this is one of the most promising sectors for AI applications (Davenport et al., 2020). Finally, we chose the UK because it is at the forefront of the digital retailing revolution (Ameen et al., 2022).

Given that AI-EP is a relatively unexplored phenomenon (Riegger et al., 2021), and the paradoxical findings that are beginning to emerge (e.g., Ameen et al., 2022), we opted for an exploratory approach. Specifically, a qualitative case study which included in-depth interviews with 18 female, millennial fashion retail shoppers, who had been exposed to a personalised advert.

The paper makes three contributions. First, we show that customers welcome this innovative way of interacting with them in the retail environment. However, their experiences with online personalisation create very high expectations of the extent of AI-EP, as well as additional services such as creation of wish lists or the ability to edit their preferences. These findings can guide practitioners’ investment in AI-EP. Second, we provide empirical evidence of how the impact of the context of message delivery, the format of message delivery and the salience of privacy concerns differs for AI-EP vs online personalisation. This can guide the application of findings from extant research, and guide future research efforts. Third, we identify the content and process gratifications derived from AI-EP, extending Sutanto et al (2013)’s work on the manifestation of the personalisation-privacy paradox among smartphone users.

The paper is organized as follows. Section 2 considers the emerging literature on the opportunities and challenges for AI use in physical retail. Section 3 presents the theoretical background. Section 4 articulates the approach to data collection and analysis. Section 5 communicates the empirical findings. Section 6 discusses the findings, and uses the motif of the Snakes and Ladders game to capture the factors that support or prevent acceptance of AI-EP, Finally, Sect. 7 captures the contributions of this empirical investigation to the advancement of theory and practice of AI deployment for personalisation in physical retail environments.

## Research Background

### Prior Studies in AI in Retail

AI studies have seen a significant amount of attention in recent years from many different disciplines, and applied to many different settings, including retail (Dwivedi et al., 2021).

Several studies propose that AI can help retailers develop new and innovative applications from the various datasets available to them (e.g., Davenport et al., 2020), and in doing so, achieve competitive advantage. However, they tend to lack empirical evidence, and to overlook the customer perspective. There is also a growing a body of work focusing on the obstacles to effective use of AI (e.g., Boratto et al., 2018). Authors mention the risk of consumer backlash and of negative impact for firms. Though, the lack of customer focused research results in insufficient understanding of consumers’ perceptions of AI use in retail.

In turn, the literature on digital personalisation (e.g., Boerman et al, 2017) suggests that AI-EP could enhance but also frustrate customers. Yet, except for Ameen et al (2022), these studies examine personalisation in controlled experiments rather than actual in-store experience. Finally, the effectiveness of personalisation efforts tends to be limited by customers’ privacy concerns (e.g., Aguirre et al, 2016). While some of these studies focus on smartphones (e.g., Sutanto et al., 2013), they provided limited insight into how customers manage the tensions arising.

Table 1 summarises the notable themes identified in the stream of literature related to AI and its use for personalisation. The right-hand column emphasises the research gaps.

**Table 1:** Selected recent studies on AI in Retail

Theme	Source	Key Claims	Gap Identified
AI Potential in Retail	Davenport et al., 2020; Huang & Rust, 2021; Kietzmann et al., 2018; Syam & Sharma, 2018	AI can offer impressive potential in terms of data processing power for innovations such as: improved market segmentation, predictive analytics, sales forecasting, and personalisation Back-end processes such as customer data management and sales basket analysis have been enhanced by AI	Studies focus on the envisaged potential of AI in retail, without empirical evidence. Extant studies appear to deduce overtly positive outcomes from applications of AI to retailers, overlooking the customer perspective
Challenges of AI in Retail	Boratto et al., 2018; Castillo et al., 2020; Crick et al., 2019; Dwivedi et al., 2021; Gardino et al., 2021; Griva et al., 2021	Many challenges exist for deployment of AI to process data efficiently and effectively, such as poor data availability, lack of skills and leadership buy-in, cost of deployment and ethical and regulatory restrictions Seasonal trends make prediction difficult and unstable, and can be dramatically influenced by a broad range of factors, as witnessed during the Covid-19 pandemic	The gap between the AI promise and reality could result in customer backlash and reputation tarnishing, which could have significant, and long lasting, negative impact for firms. Yet, not many studies focus on consumer perceptions
Digital Personalisation	Ameen et al., 2022; Boerman et al., 2017; Riegger et al., 2021; Sutanto et al., 2013; van de Sanden et al., 2019; Wirtz et al., 2018	Personalisation can impress as well as frustrate customers, who are seeking offers unique to them, as derived by AI	Studies examine personalisation in controlled experiments, outside of the shopping environment, and have yet to examine the in-store experience
Privacy	Aguirre et al, 2016; Awad & Krishnan, 2006; Castelo et al., 2019; De Bruyn et al., 2020; Grewal et al., 2016; Riegger et al., 2021; Sutanto et al., 2013; Yoganathan et al., 2021	Customers feel uncomfortable about, or react negatively to, interacting with AI, as it demands customer information to be effective. Customers are willing to submit information to take advantage of offers, but are ultimately uneasy with the process	Studies to date identify tensions related to AI-enabled personalised offers delivered to their smartphones, but have not gone to the extent of understanding how customers manage any tensions that may arise

### Personalisation-Privacy Paradox

The review of the literature revealed a lack of customer focused, evidenced based understanding of how AI-EP benefits retail customers, and which factors may create resistance to acceptance of AI-EP or destroy value for customers. While personalisation can bring benefits to consumers, they may resist personalisation if they deem that the collection and use of personal data that underpin personalisation is too invasive (Moore et al., 2015). This tension has been termed the Personalisation-Privacy paradox.^1^ To unpack the conditions under which the personalisation-privacy paradox manifests in each context, it is necessary to identify the gratifications that users derive from interacting with the medium through which personalisation is delivered, as well as their desires and concerns about information privacy (Sutanto et al., 2013).

#### Gratifications from Personalisation

Sutanto et al (2013) put forward two types of gratification arising from personalisation: *content* gratification, referring to the enjoyment derived from the personalised message itself; and *process* gratification, referring to the enjoyment derived from the medium in which the personalised offer is delivered.

The personalisation literature identifies various content related gratifications such as receiving offers that reflect customers’ preferences (Krishnaraju et al., 2016; Pappas et al, 2017) and context (Xu et al., 2011), reducing the effort or time required to complete the purchase (Tam & Ho, 2006), and enabling cost savings and other financial gains (Schmidt et al., 2020). However, personalised messages can also stir negative emotions such as irritation (Haghirian et al., 2005) or anger (Pappas et al., 2018), thus rendering personalisation efforts ineffective (Demoulin & Willems, 2019). Customers are likely to resist offers that are seen as a threat to their freedom of choice (Brehm & Brehm, 2013). AI-EP may be perceived as restricting the options available to customers, which may result in customers rejecting the AI offer, in order to reaffirm their autonomy (André et al., 2018).

In turn, process gratification arises from the ability to control how messages are received (Brusilovsky & Tasso, 2004), such as being able to filter out certain messages, or to control when and how they are displayed (Sutanto et al., 2013). Research has also shown that being able to control which information is collected and how it is used increases message effectiveness (Tucker, 2014), while lack of transparency from firms has the opposite effect (Aguirre et al., 2015). AI algorithms are, typically, opaque (Burrell, 2016), preventing customers to see – and influence – how they produced a specific recommendation, which may result in resistance to AI-EP.

While Sutanto et al (2013) found, in the context of smartphones, that personalisation gives users process gratification but not content gratification, by and large, the personalisation literature focuses on the latter (Boerman et al., 2017).

#### Privacy Concerns

The effectiveness of personalisation efforts may be offset by users’ concerns over the privacy of their personal information (Awad & Krishnan, 2006). For instance, online ads that closely match customers’ browsing history reduce purchase intentions, because they raise concerns over firms’ surveillance practices (Aguirre et al., 2016). Customers set boundaries – psychological or physical – around their personal data (Stanton & Stam, 2003), and attempts to cross those boundaries raise concerns, and are met with resistance (Xu et al., 2008). Customers manage information boundaries by selectively sharing or withholding information (Sutanto et al., 2013). In addition, they may purposefully provide false information, such as using a false name or birth date (Miltgen & Smith, 2019), when firms attempt to collect personal data that they deem private.

The literature indicates that customers may be comfortable disclosing information deemed to be relevant for the intended outcome (Xu et al., 2011), when access to the service is time critical (Hubert et al., 2017), and where the information is routinely requested in that context (Stanton & Stam, 2003). However, customers resist sharing information that is deemed sensitive, such as their health status (Sutanto et al., 2013); or which could be used for discrimination (Stanton & Stam, 2003). They also resist sharing information when they feel that they lack control over what data are collected, how data are used, and with whom they are shared (Liu et al., 2019; Schmidt et al., 2020). However, information boundaries vary across individuals and are dynamic. Namely, those customers that value information transparency are also most likely to resist the data collection that underpins personalisation (Awad & Krishnan, 2006). Customers also change whether they share information depending on the perceived gains or losses of each situation (Kar, 2020). The perception of being under surveillance is particularly prevalent in online interactions and in smart services (Bues et al., 2017).

Therefore, in addition to providing privacy features (Awad & Krishnan, 2006), firms also need to identify which information customers are comfortable to share, and what trade-offs they are prepared to make in order not to break their personal information boundaries (Pentina et al., 2016). This is particularly relevant for AI-EP, given the need for large volumes of data to support the development of targeted offers (Davenport et al., 2020).

## Research Design

The aim of our study was to advance the conceptual understanding of AI-EP by investigating the following research question: “How do consumers experience and respond to AI-EP?”. Hence, a qualitative, exploratory case study methodology (Sarker et al., 2018) was adopted. The unit of analysis was shoppers’ interactions with an AI-enabled smartphone application, in the context of fashion retail. This methodology offered an opportunity to collect primary data from customers in situ experiencing the AI-enabled personalisation offer, guided by key studies in the field (e.g., Ameen et al., 2022; Riegger et al., 2021). It also offered the unique opportunity to collect rich and diverse perspectives from participants, as they reflected upon the hybrid digital-physical experience of AI-EP, extending previous works in the area, particularly Sutanto et al. (2013). In doing so, the method adopted allowed us to understand and analyse a broad range of participant views, and to theorise and conceptualise (Eisenhardt, 1989), in line with other case studies that have examined the impact of technology upon personalisation (e.g., Griva et al., 2021).

### The Selected App

**Fig. 1:**
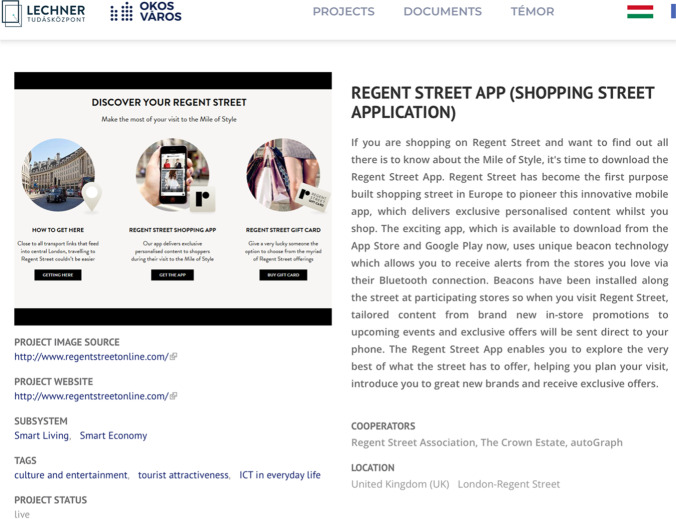
Case study App. **Image source:** http://okosvaros.lechnerkozpont.hu/en/node/558

The mobile app selected as the focus for this case study was the Regent Street App. The app was first launched in 2012 to enhance the shopping experience of visitors to this famous shopping district, in London (UK). As shown in Fig. 1, the app included the option to receive personalised offers while shopping in the area. To create and deliver these offers, the app combined “*two technologies: geofencing beacons that use location aware to offer content to users within a specified proximity to the store and cloud-based artificial intelligence (AI) to ensure personal relevancy of offers*” (Lemmon, 2017).

Circa 80% of the stores in this shopping district joined the scheme, implementing the associated technology in their premises, such as beacons around the store and microchips in the items on sale (Scott, 2014), in addition to artificial intelligence programme to personalise the offers. Moreover, 98.6% of app users created a personal profile and signed up to receive personalised content (Lemmon, 2017).

The AI-EP messages are delivered when app users are in the vicinity of the stores that signed-up to the app (Dempsey, 2015), resulting in a 7.4% increase in response rate for AI-EP vs. untargeted offers (Lemmon, 2017).

### Data Collection

To gather customer experiences, we used in-depth, semi-structured interviews, to allow participants to articulate their actions and intentions towards the AI-EP, as well as implications for their personal data.

**Fig. 2:**
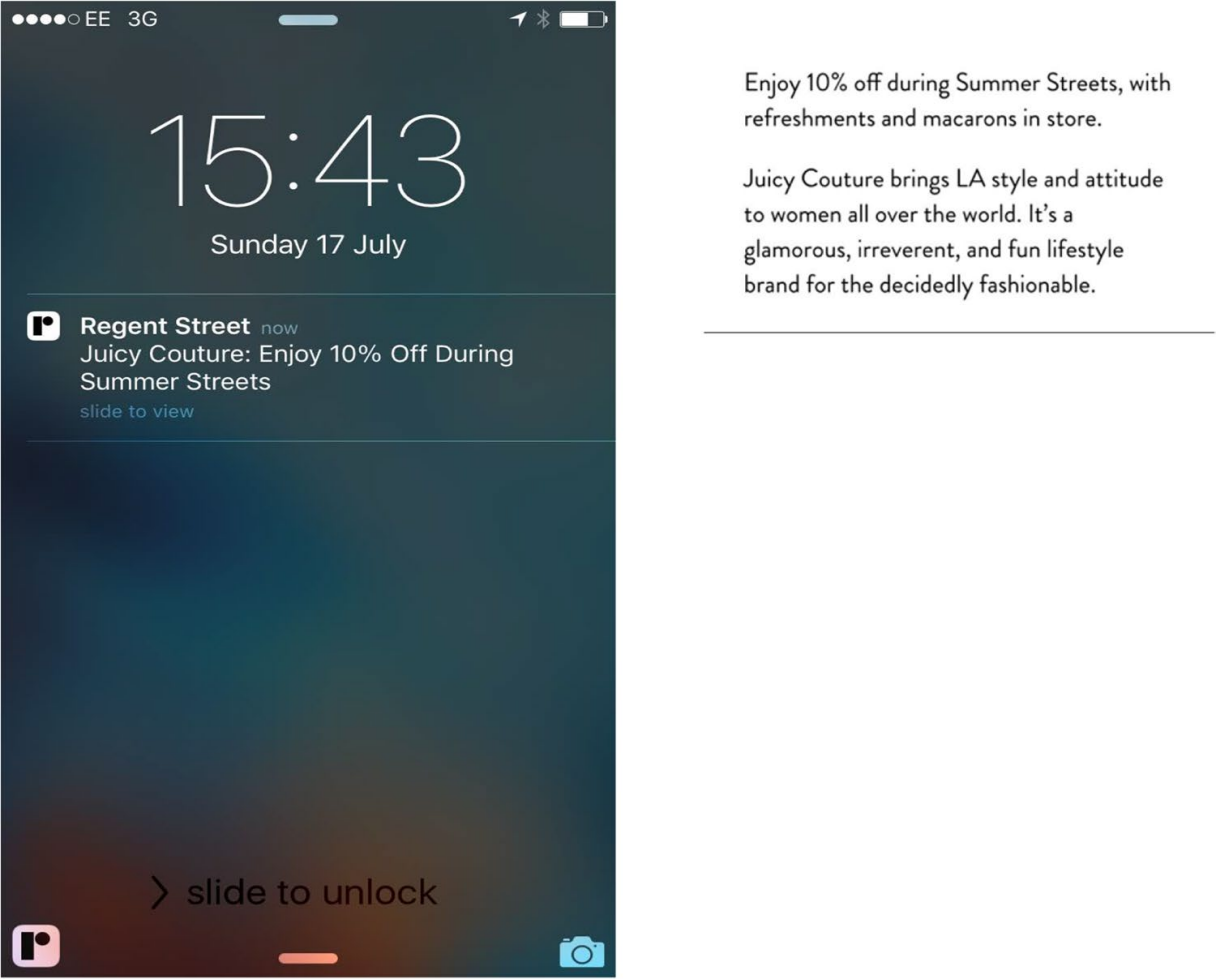
Interview prompt

In order to recruit participants, one of the authors (who conducted all the interviews) positioned themselves outside a specific fashion store in Regent Street, which was known to use the Regent Street App for the delivery of AI-enabled personalised offers. As shoppers walked past the store, the interviewer approached them, showed them the advert in Fig. 2, and invited them to participate in an interview. This approach is in line with Kar (2020)’s recommendation that research on customer perceptions of digital technology should take place immediately after encounter with that technology.

Some interviews took place outside the store, others at a nearby café. No financial incentives were offered to the interview participants. The interview protocol (Table 2) reflected the key themes identified in the extant literature. The questions focused on perceptions of the message rather than the technology underpinning it, as customers don’t always understand the technology behind personalisation. This approach allowed us to move beyond a simplistic view of positive vs. negative attitudes, and to understand the black box of the customers’ response (Belk, 2017).

**Table 2:** Interview protocol

Section	Question
Stimulus	*Participant receives targeted prompt. Upon opening the screen, the participant learns that the offer is exclusive to users of the Regent Street mobile app walking past that store, and who have bought in that store, previously* 1. What do you think of this offer?
AI-EP – Gratifications	2. This offer has been personalised based on your location and shopping preferences. Is this offer useful? 3. How does it enhance your shopping experience? 4. When the brand sends real-time, relevant offers to your mobile phone, are they mostly trying to sell more, or trying to build relationships with customers like you, by serving your specific needs? 5. Do you think that the company will always make the best offer specifically for you? 6. Why do you suppose that?
AI-EP – Outcomes	7. Do personalised offers help you develop bonds with this brand? 8. Would receiving this type of offer discourage you from switching to another brand? Why?
AI-EP – Privacy concerns	9. Which personal information would enhance your experience with this retailer? [Probe for location and behavioural data] 10. Are you willing to share that information with the company, so that they can develop offers specifically for you? 11. Where should the limit be? 12. What are the benefits of letting the company access your personal data? 13. What are the risks of letting the company access your personal data? 14. Through the app, the company can track your movements not only in-store but also in the proximity of stores on Regent Street? How does that make you feel?

As resistance to AI-EP may depend on customer characteristics (Yoganathan et al., 2021), we recruited an homogeneous sample via purposive sampling (Bryman & Bell, 2015), to give direction to the data collected in support of the case study (Yin, 2012). We focused on female shoppers aged 18 to 30 years old, because, in the UK, this demographic group cares the most about looking trendy (YouGov, 2020). Women in this age group are twice as likely than men to agree that they spend a lot on clothes and to value immediate access to fashion items; and they are also more likely than men and then older women to shop at multiple retailers (YouGov, 2020). Consequently, this demographic group are a key target for high street fashion retailers’ promotional efforts. This demographic group are also more open than others to sharing their personal data with firms, given that they grew up in a digital world (Liu et al., 2019). However, women may resist AI, especially when outcomes are consequential (Castelo et al., 2019). We conducted 18 audio-recorded interviews, each lasting between 30 min and one hour. Each recording was transcribed with an average of 9,000 words, equating to just over 160,000 words in the final dataset. The data was checked for accuracy and prepared for analysis.

### Data Analysis

The interview data was analysed using NVIVO and following Krippendorff's (2004) systematic approach to thematic analysis. As is customary of exploratory case studies in the information systems discipline (see Sarker et al., 2018), the theory was used to guide the design of the study and to set the general direction of data analysis. In practice, this meant that a preliminary coding book was developed based on the themes identified in the literature, and this was used in stage 1 of data analysis to deductively code the transcripts into a) gratifications from personalisation, b) privacy concerns and c) reaction to AI-EP. Subsequently, in stage 2, for each of the themes in the code book, the analysis of the data proceeded in an inductive fashion, with subsequent codes emerging from the data. The final set of codes is depicted in Table 3.

**Table 3:** Coding structure

Aggregation	1^st^ order	2^nd^ order	Illustrative quotes
Gratifications	Content	Relevancy of the offer that is better than humans	*I mean if you can choose what you like and then they will remember it that would be so much easier to go and shop there and maybe you would buy a bit even more*
		Time saving attributes to the customer experience	*It’s really useful because you get to know what is there*
		Financial benefits that are attractive to modern customer base through appetite for discounts	*I would value it a lot. There is nothing to lose for customers and it is not like we are committing to a sale of any sort or a purchase of any sort*
		Other benefits	*But also the things like pretty macarons or lemonade*
	Process	Message Delivery	*If I would receive an offer from a store I really like and I already have a 10% offer, I would definitely go inside and check out the stuff they have*
		Information collection process	*I would rather have a setting in application—right now I am shopping for my dad. Rather than registering it under me. Or buying gifts for him or for her rather but still that the information being given*
		Information use processes enhancing value to the in-store experience	*It would definitely help because I can make a profile of things I like. It is an amazing tool definitely*
Privacy concerns	Information boundaries	Boundary management practices	*I only share information about fashion. Only information where I know it can create value for me*
		Information—Willingness to share	*if somebody wants to track me down they can do it, they have (the data), anyway… but on the other hand, it does not really matter what they are going to do because they can have it anyway*
		Information—Desire to protect	*I want to know if it’s going to be used for more than just trying to fulfil my needs within the shop*
Acceptance of AI-EP	Perceptions	Positive	*Sometimes I just want to have something which I already have, which is different from what I already have. So, personalizing is useful for me in terms of fashion*
		Negative	*If the company has bad intentions, there may be some downside in sharing the information*
	Behaviour	Acceptance of AI-EP and Customers are willing to share information to receive personalisation offer	*Telling them about your style, so they would know what specific things to target to you, and maybe saying your age group and gender, because that might help them to target you towards particular things as well*
		Rejection due to irritation from notifications, interruptions, lack of control	*If I am not shopping I would not want that sort of notifications or if I am doing something else I do not know. If you end up passing there every day it could be quite annoying*

The findings emerging from this analytical process are presented in the next section, following a polyphonic account. This approach presents the range of perspectives offered by the research participants in order to develop a layered account of the phenomenon being investigated (Travers, 2001), as is customary of interpretive research. This is in contrast with identifying the dominant narrative or single shared reality typical of positivist approaches to data analysis (Sarker et al., 2018).

## Findings

### Gratifications from Personalisation

Our participants were very positive about using the app while shopping in Regent Street and receiving personalised recommendations on their phones: “*You are going to (Regent Street) in your free time, and want to have a nice day, and, through, the app it might be even nicer*.” (Interviewee 3).

Contrary to the participants in Sutanto et al (2013)’s experiment, for whom personalisation via smartphone apps delivered process but not content gratification, our participants identified both types of personalisation. The analysis of the data (Table 4) showed that the participants experienced relevance, time savings and financial gratifications, in line with the literature on online personalisation. However, for most of our interviewees, discounts seemed to be the main benefit expected from AI-EP, undermining the promise that this form of personalisation can increase basket variety and improve retailer profitability (Kumar et al., 2017). For those interviewees, discounts might be complemented by other benefits, such as time savings, but not replaced by them.

**Table 4:**
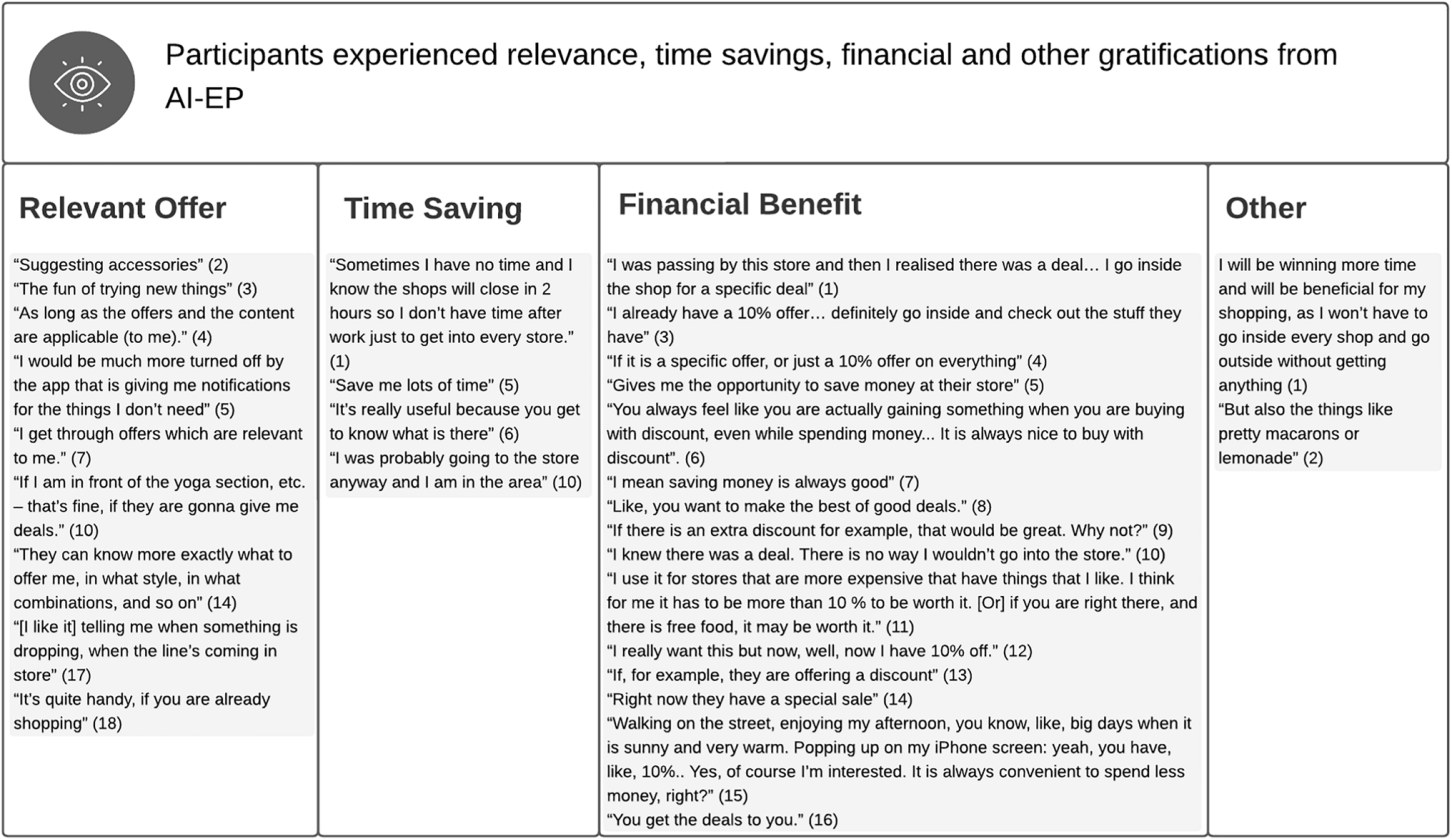
Content Gratifications

As for process gratifications (Table 5), some deemed receiving personalised notifications on their phones as superior to relying on shop window information to gain information about new products or about deals (e.g., participant 11); or receiving offers via e-mail (e.g., participant 16). However, many more commented that, at times, the volume of notifications became a nuisance. This is particularly relevant for the Regent Street app, as this is a central London location, next to theatres, cafés and other leisure venues, as well as a commuting route, as mentioned by participant 4. A high volume of notifications could result in information overload (e.g., participant 13), intrude in relaxation time (e.g., participant 6), as well as drain the phone’s battery (e.g., participant 18). While most participants mentioned the option of switching off their Bluetooth to stop notifications, this was an unsatisfactory solution for many. Instead, many expressed the desire to control effortlessly when to receive notifications and what type, which is in line with literature on the role of customer autonomy in technology interactions (e.g., André et al., 2018).

**Table 5:**
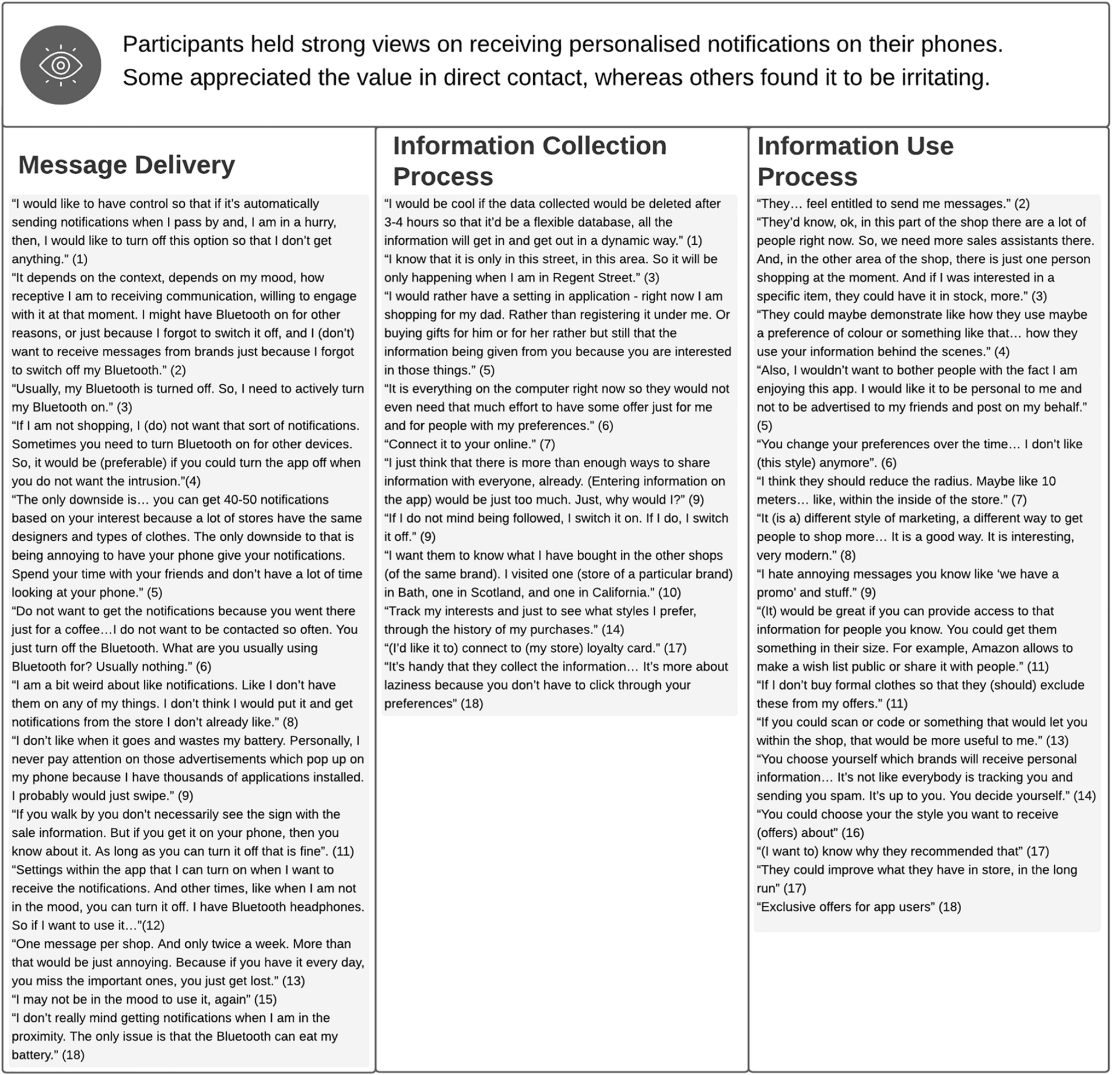
Process Gratifications

Opinions varied as to whether the app was an effective way of collecting and using information for AI-EP. Some, like participant 14, were happy with the data collection process. However, others felt that the app should integrate with other data sources (e.g., participant 7). Still, others, like interviewee 5, lamented the lack of ability to edit purchase histories, or to select when not to collect data (e.g., for gifts and other one-off purchases). Because AI doesn’t understand the reasons behind a purchase (Woo Kim & Duhachek, 2020), the research participants predicted that one-off purchases would be added to their purchase history, undermining the quality of future recommendations. Two interviewees (4 and 17) indicated an explicit desire to understand why they had received specific recommendations. In Sutanto et al (2013)’s examination of app users’ willingness to share personal information, the process benefits referred to the experience with the medium itself (namely, navigation of the app). However, interviewees 3 and 17 also seemed to value process benefits at the level of the in-store experience broadly, emphasising the hybrid nature of AI-EP.

### Privacy Concerns

In terms of boundary management behaviours, as detailed in Table 6, we found various instances of selective information disclosure to tap into benefits. For instance, the interviewees were willing to provide information directly into the app or via surveys (e.g., participant 9) to improve the accuracy and relevance of the resulting recommendations. They also engaged in redemptive behaviours (Stanton & Stam, 2003), whereby they shared information to reduce the losses generated by irrelevant recommendations, as illustrated by Interviewee 5. The interviewees were also keen to engage in information withdrawal. In particular, they wanted to remove records of one-off purchases, as well as historical information that was no longer relevant (e.g., participant 6), corresponding to Stanton and Stam (2003)’s political and protective behaviours. However, those options were seen to be unavailable or too difficult to access. Finally, we did not find evidence of interviewees disclosing fake information to manage the benefits and risks of AI-EP, contrary to what was reported in Miltgen and Smith (2019).

**Table 6:**
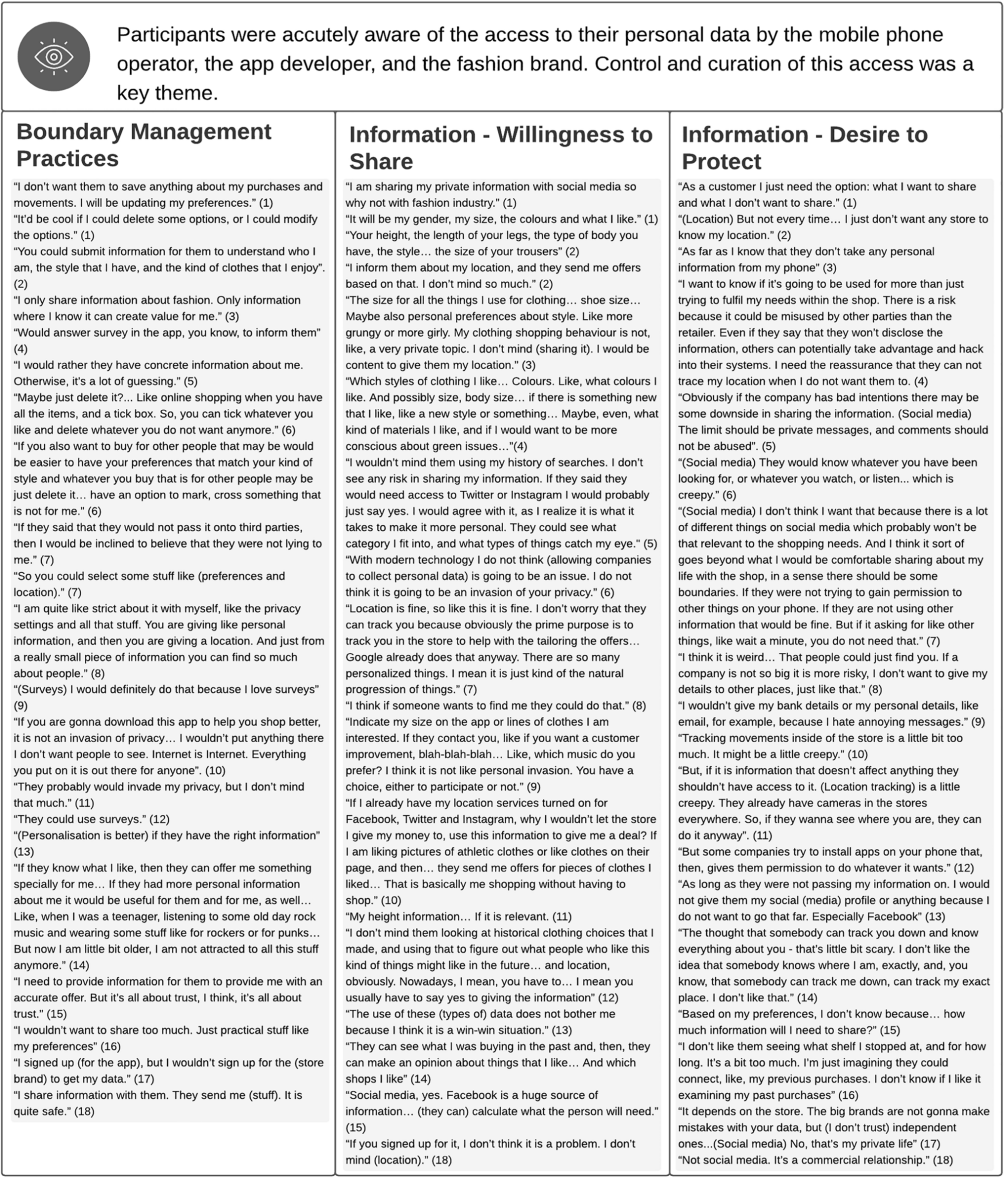
Privacy Concerns

The interviewees were aware that, by using the app, a range of companies could access their personal data, including the mobile phone operator, the app developer, and the fashion brand. This situation was seen as the default in the digital era, as illustrated by interviewee 6’s quote. As shown in Table 6, an in line with extant literature (e.g., Miltgen & Smith, 2019), the interviewees were willing to share information such as clothes’ size, specific body measures, preferred styles, or favourite colours, to obtain relevant recommendations. As Interviewee 13 said: “*The use of these (types of) data does not bother me because I think it is a win–win situation*”. In contrast, and in line with Sutanto et al. (2013), most were unwilling to share personal information which they did not deem essential for the task at hand, or which could leave them vulnerable to manipulation, nuisance, or fraud (e.g., Interviewee 4).

The topics of location and social media data divided opinions, however. Regarding the former, interviewees 3, 12 and 15 expressed the view that sharing geo-tracking was a natural extension of what already happens on other media and was useful to develop targeted offers. However, the others expressed reservation towards various aspects of the tracking of their location. They described this activity as “creepy” (e.g., interviewee 11) and, in line with Schmidt et al. (2020), they expressed a strong desire to limit the app’s ability to track their movements (e.g., interviewee 2). Regarding social media data, interviewees 1, 5 and 15 were in favour. But the remaining felt that these data should be off limits to the app. Some, like interviewee 6, rejected this because they felt that the data would be too revealing; others, like interviewee 7, because social media data were deemed irrelevant.

Two key nuances emerged regarding privacy concerns associated with AI-EP. The first nuance relates to control over access to personal information. Specifically, interviewees would be willing to share more information if they could be in control of what data was collected and when (e.g., interviewee 1), or if they were reassured that the app provider would not take advantage of the situation to access other areas of their phones (e.g., Interviewee 3). The second nuance refers to trusted parties. The app provider was, implicitly, a trusted party, but this sentiment did not necessarily extend to specific stores on the app, particularly smaller ones (see Interviewee 17) due to concerns of the latter’s ability to fend off security attacks. On the other hand, there were other parties that the interviewees trusted more than the app provider – namely, Apple (as mentioned by interviewee 10).

### Acceptance of AI-EP

The literature’s enthusiasm for AI-EP (e.g., Bues et al., 2017) was mirrored in our research participants’ reactions. The analysis of the findings (Table 7) reveals that some participants found this type of offers interesting (e.g., participant 8), exciting (e.g., participant 1) and useful (e.g., participant 17). Many felt valued by the company behind the offers (e.g., participant 10) and, as a result, developed a positive attitude towards the company (e.g., participant 3), which indicates the potential of AI-EP for relational benefits (Liu et al., 2019). Having said that, 10 out of the 18 participants could not see any relational benefits. They expressed scepticism about the intentions behind AI-EP offers, seeing them as mostly an attempt to get customers to increase their expenditure (e.g., participant 12). Participant 4 also expressed scepticism about AI-EP’s ability to meet her needs, due to limitations of the technology, as well as the associated cost. Other negative emotions reported were annoyance (e.g., participant 16), and creepiness or the feeling of being stalked (e.g., participant 9). Some participants also reported a feeling of intrusion in what is meant to be a leisurely, relaxing activity, with interviewee 8 describing it as thus: “*It’s like a sales assistant running out into the street and grabbing me.*” In addition, interviewee 15 reported a fear of over-spending as a result of AI-EP, while participant 18’s comment that “*You would be less aware of what is going on. You would be in a loop*” echoes the perceived threat to freedom of choice identified by Brehm and Brehm (2013).

**Table 7:**
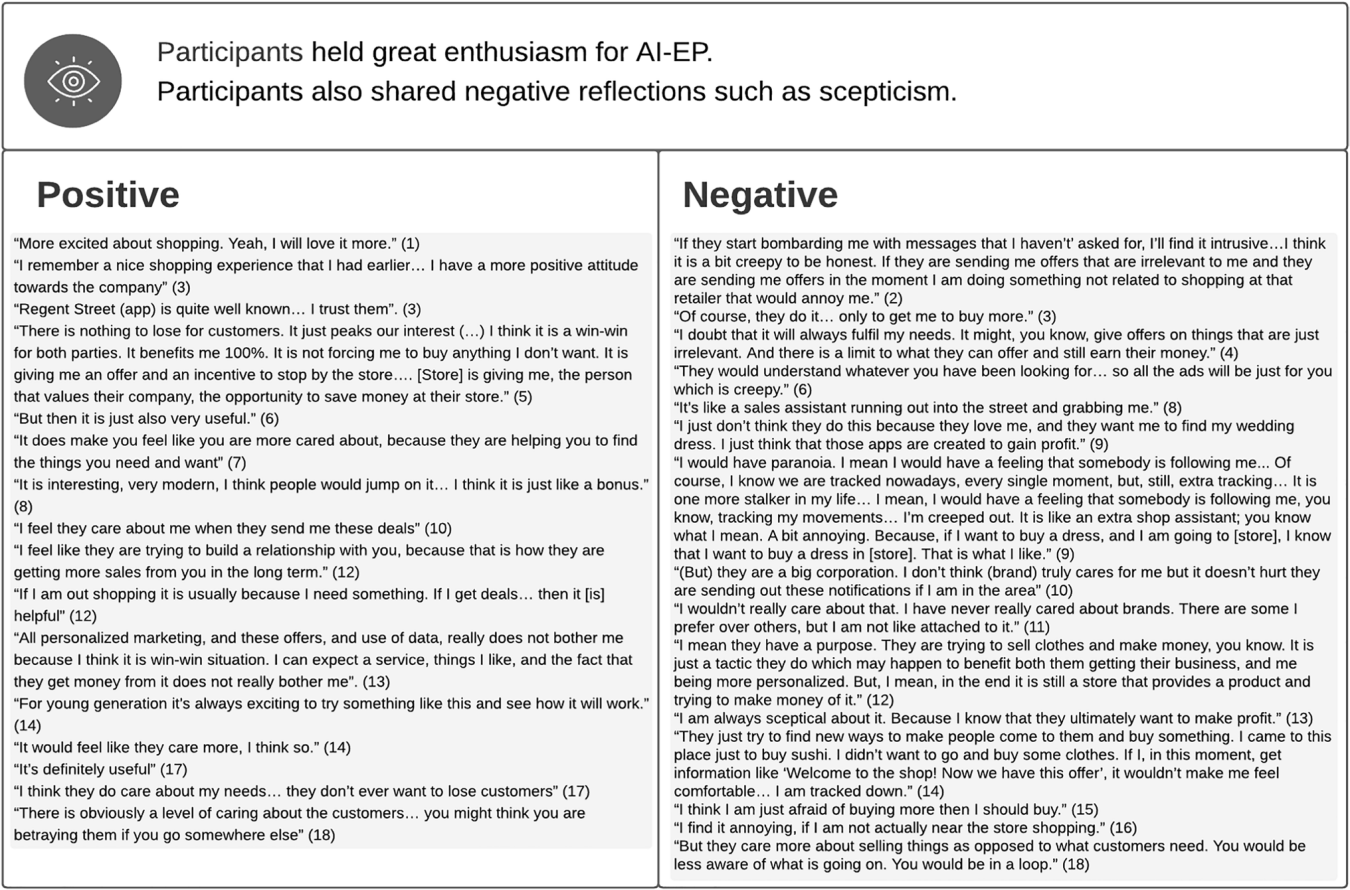
Perceptions of AI-EP

The positive sentiments translated in willingness to act on the offers delivered via AI-EP, particularly if they came in the form of exclusive, time-limited discounts, for their favourite stores, as exemplified by participant 3’s quote (Table 8). While participants 13 and 17 said that AI-EP might lead them to try new stores, most ignored offers from stores that they did not usually shop at, or which they were not familiar with. That is, it seems that AI-EP works better for customer retention than for customer acquisition, and for the pre-approach stage of the sales process, which contradicts claims that AI can add value at any stage of the sales process (e.g., Syam & Sharma, 2018).

**Table 8:**
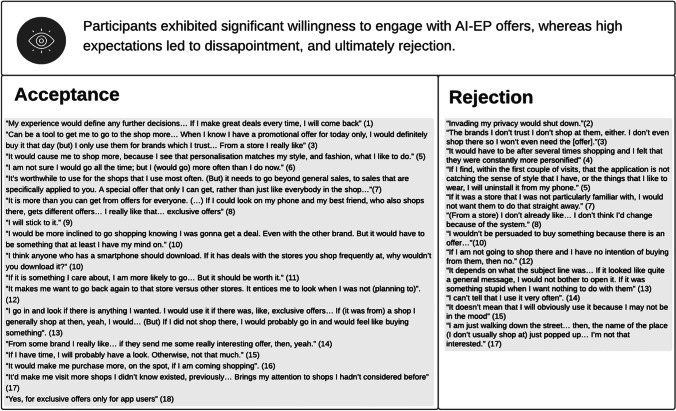
Behavioural Outcomes

However, participants have very high expectations of AI-EP. While some are willing to accept some trial and error (participant 4), in general, they expect extremely targeted and unique offers (e.g., participant 8). This expectation might reflect the participants’ experience with personalisation in the online environment, where users typically receive very unique recommendations (Griva et al., 2021). Failing to meet such expectations seems to result in disappointment with the app (e.g., participant 5, Table 8), rather than with the brand (e.g., participant 4, Table 7). This reaction is in contrast with extant literature on online personalisation (e.g., Baek & Morimoto, 2012), but aligned with literature on mobile shopping apps (e.g., Shankar et al., 2016).

## Discussion

The extant literature argues that fashion retailers may enhance the customer experience through the use of AI-EP by harnessing company-owned as well as external datasets to create highly individualised offers (van de Sanden et al., 2019). Though, the broader personalisation literature implies that the effectiveness of AI-EP may be compromised by privacy concerns (Aguirre et al, 2016), and that AI-EP may even result in customer dissatisfaction, due to inflated expectations or negative experiences (e.g., Karumur et al., 2018). Our focus on the customer perspective, and the exploration of an actual in-store experience, provides empirical evidence of the tensions in place, and how customers navigate them, as discussed next.

### The Personalisation-Privacy Paradox in the AI-EP Context

Our findings are aligned with those from research on personalisation in the online environment, which established that personalised offers may deliver content gratification in the form of relevance (Krishnaraju et al., 2016), plus time (Tam & Ho, 2006), and cost savings (Schmidt et al., 2020). Though, in AI-EP, the opportunity for cost savings seems to dominate over the other forms of content gratification mentioned in the online personalisation literature.

The limited importance of relevance in AI-EP might reflect the nature of shopping in the physical environment where, typically, there are fewer options on display than in online shopping (Kumar et al., 2017). Therefore, customers may feel less overwhelmed by choice in the physical environment. Moreover, some of our interviewees seemed to associate fashion shopping in the physical environment with an hedonic experience (Gardino et al., 2021), rather than a functional one. The pleasant nature of in-store shopping may explain the reduced importance of time savings in AI-EP vs. online personalisation. The resistance to suggestions by the AI could also indicate that customers do not trust that AI has the skill to make such recommendations (Woo Kim & Duhachek, 2020), given that fashion shopping is a task rich in intuition and subjectivity (Castelo et al., 2019).

This familiarity with personalisation in online fashion retail suggests a compelling path for future adoption by retailers. However, the emphasis on discounts contradicts the prediction that AI-EP will generate additional sales opportunities and improve retailer profitability (e.g., Kumar et al., 2017) by prompting customers to consider complementary items, or generating impulse purchases (e.g., Griva et al., 2021).

Our findings also show the need for a careful approach to the process of delivering the personalised offer. In line with previous research on personalisation online (e.g., Brusilovsky & Tasso, 2004) and on smartphones (Sutanto et al., 2013), many participants expressed a strong desire for controlling notifications and other aspects of message delivery. Moreover, we observed intricate interactions between the receipt of notifications and various contextual factors such as phone battery depletion, the purpose of visit (e.g., shopping vs meeting friends) or additional information provided.

Customers also expressed a strong desire to be in control of the information held in the system and used to create personalised recommendations, which is line with findings from online personalisation research (e.g., Aguirre et al., 2015; Tucker, 2014). Moreover, customers wanted the ability to edit information held by the retailers and which they perceived to be undermining the quality of the AI-EP. However, it is not clear that enabling customers to engage in such boundary management behaviours (Stanton & Stam, 2003) would deliver the results sought by retailers. As shown in the context of online personalisation, messages need to be persuasive in order to be successful (Pappas et al, 2017); and fashion retailers need access to large and stable datasets about customers and their context (Ameen et al., 2022) in order for the AI to create high quality, persuasive messages.

Some customers also expressed a desired to understand why they received specific recommendations. It will be difficult for retailers to meet this particular customer expectation because algorithms are opaque, and it is difficult to trace exactly which data inputs are generating which outputs (Burrell, 2016). As a result, some customers may reject the AI-EP offer to reaffirm their autonomy (André et al., 2018).

Exposure to widespread collection of personal data in the online environment may have influenced our respondents’ willingness to share data for AI-EP (Stanton & Stam, 2003). Many also showed willingness to participate in ad-hoc data collection initiatives, as they saw these as an opportunity to improve their shopping experience. However, there were noticeable nuances in terms of comfort with disclosing certain types of personal data, which require a very careful approach from retailers in order not to violate customers’ personal information boundaries (Pentina et al., 2016). Mobile apps are useful tools to collect data such as unique customer identifier and transaction history, due to the high penetration of mobile phones, and because they can be linked to individual users (Shankar et al., 2016). However, customers need to perceive a link between the information requested and the resulting offer (Xu et al., 2011). Moreover, firms need to avoid collecting information which customers deem likely to be misused, or to leave them in a vulnerable position. Some participants also opposed the collection of social media activity.

Another data input that is essential for instore AI-EP is location (Schmidt et al., 2020). This can either be individual data such as the customer’s whereabouts, or contextual data such as the weather or crowd levels (Verhoef et al., 2017). However, the emotionally charged descriptors used by some of our participants, indicate that customers intensely dislike extensive tracking in the physical environment. This presents a challenge for fashion retailers: one the one hand, location data enables them to take full advantage of AI’s capabilities for personalisation; on the other hand, customers may see this as an invasion of privacy (Xu et al., 2008), which may result in negative attitudes towards AI-EP and, ultimately, its rejection (Shankar et al., 2016).

### Effectiveness of AI-EP

Based on our findings, attempts to use AI-EP for customer acquisition may be ineffective (Demoulin & Willems, 2019), or even detrimental (Baek & Morimoto, 2012) for the brand. This finding was somehow surprising given that the app considered in this case study was provided by a trusted party which offered discounts to a variety of stores in a given shopping district. Trust has been shown to impact the perception of a personalised offer (Aguirre et al., 2016) and, as such, familiarity with the Regent Street app might lead customers to be receptive to AI-EP attempts from new brands (Chen & Dibb, 2010).

Furthermore, we found that customers expressed a strong desire for autonomy and freedom of choice, as reported in the context of online personalisation (Balan & Mathew, 2020). Though, while previous research focused on choice and agency in relation to the content of the message, we witnessed a willingness to control message delivery, too. Granting this flexibility might return a sense of control to customers (Brehm & Brehm, 2013), but may increase the complexity of the app (e.g., in terms of navigation), which will negatively impact the user experience (Shankar et al., 2016). Moreover, it reduces the retailers’ ability to collect data and deliver targeted messages (Chou & Shao, 2021).

While AI can integrate multiple sources of customer, contextual and transactional data, our study exposes limitations to the extent of in-store personalisation (Ameen et al., 2022; Boratto et al., 2018). Namely, in contrast with the online environment, where personalisation may influence the search and evaluation stages (Davenport et al., 2020), AI-EP was revealed to be most valued at point of purchase stage, albeit not for payment purposes. Furthermore, whilst algorithms underpinning AI-EP need to be rigorously tested (Sutanto et al., 2013), our findings indicate that fashion shoppers have low tolerance for such trial and error. As in the online environment, consumer trust and positive emotions are essential for successful personalisation (e.g., Pappas, 2018). As with personalisation in the online environment (e.g., Pappas et al, 2017), customers have high expectations of AI-EP. The inflated expectations and the low tolerance for mistakes, are likely to result in disappointment and app abandonment (Riegger et al., 2021; Shankar et al., 2016), represents a waste of resources, and inability to continue collecting data about customers.

**Fig. 3:**
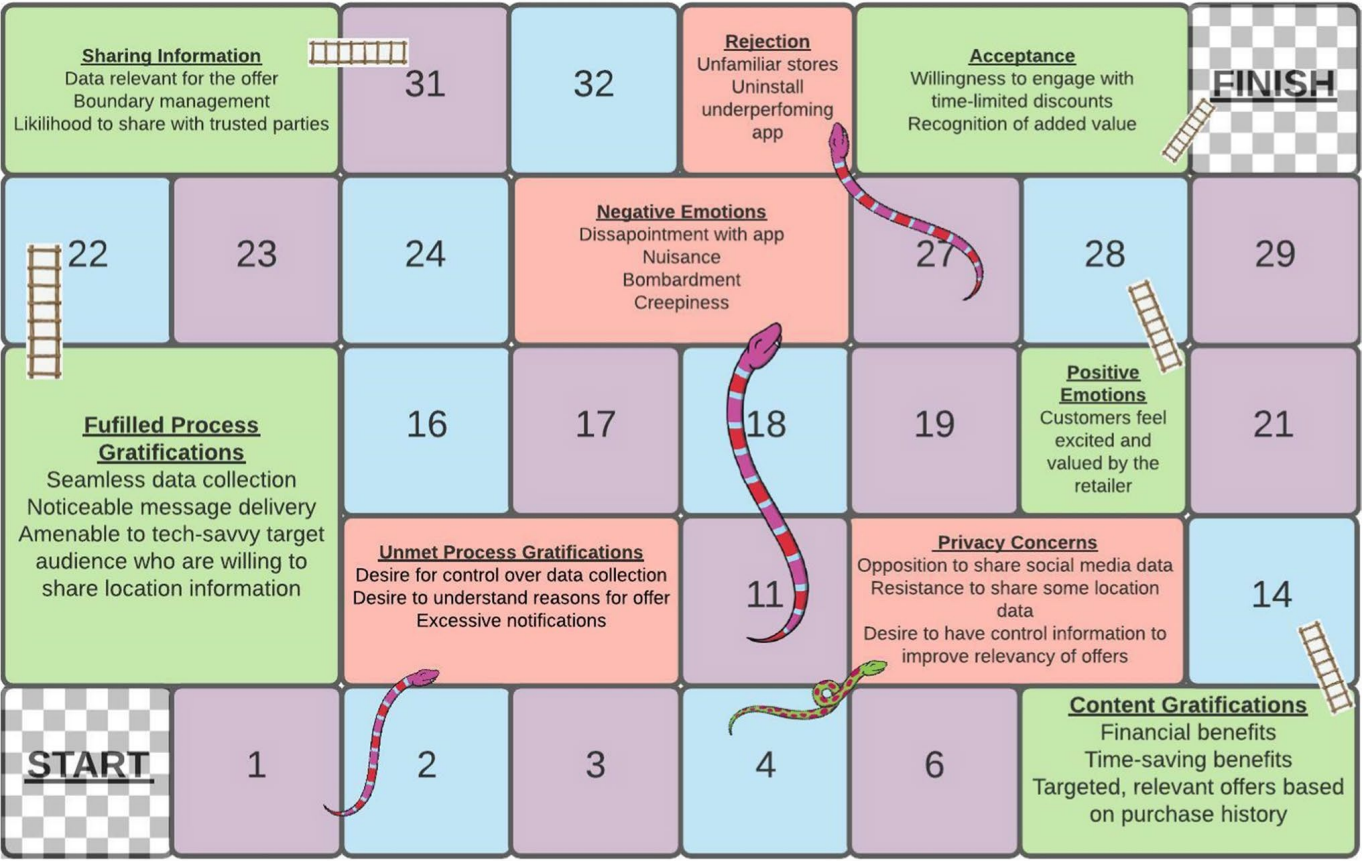
The Snakes and Ladders of AI-Enabled Personalisation

Figure 3 presents an overarching view of how in-store AI-EP can enhance customer experiences, capturing both the enabling factors from content and process gratifications, and the detracting factors related to unmet process gratification expectations and from privacy concerns. We represent the AI- EP journey consisting of opportunities and threats for retailers, as encapsulated in the well-known game of Snakes and Ladders. This model highlights the potential as well as the risk for brands about to embark upon such an endeavour. Moreover, from our review of personalisation in both retail and digital spheres, this is the first such conceptual framework of its kind representing the user-end perspective of such innovations in technology.

The game begins from the moment a user/player is within proximity of the store. The player is then faced with two options, either an enabling force (indicated by a ladder) moving them higher up the personalisation journey, or a detractor (indicated by a snake) preventing progress on the board. Each factor is described with key attributes as generated from the findings of the study. We envisage that AI-EP is not a one shoe fits all experience for users, and that it may take a circuitous route. As retailers continue to innovate, the blank squares represent the stages of the journey not relevant to AI-EP. The final goal is where the AI-EP has delivered a positive in-store experience and created value for customers and retailers.

## Conclusion

The deployment of AI technology for personalisation promises to address some of the business challenges faced by high-street retailers (Kumar et al., 2017), such as increased competition, heightened price sensitivity or the emergence of the show-rooming phenomenon. AI-EP apps, such as the one analysed in this paper, enable the creation of offers that draw on individual behaviours and contextual information, as opposed to aggregate segment information (as in the case of non-AI, automated personalisation) or intuition (as in the case of sales staff personalisation). As a result, AI-EP offers can be more relevant, granular and timely than either of those alternatives. However, factors related to the context of message delivery, the format of message delivery, and the salience of privacy concerns may impact the relevance of extant research on technology-enabled personalisation—mostly performed in the online environment—to help us understand consumers’ acceptance of AI-EP. Therefore, we responded to calls by Ameen et al (2022), Riegger et al (2021) and van de Sanden et al (2019), among others, for empirical research on how consumers experience and respond to AI-EP.

The qualitative investigation of consumers’ interaction with AI-DP in a shopping district with London, UK, through the lens of the personalisation-privacy paradox enabled us to identify the perceived content and process benefits derived from AI-EP, as well as how privacy concerns undermine these benefits and inform the customers’ boundary management tactics. Together, these factors result in a carefully orchestrated process whereby customers either accept or reject the artificial intelligence-derived personalisation offer, but with a high degree of control over their interaction with the offer, and in particular the use of their personal information.

### Theoretical Contributions

Our study makes the following three contributions. First, we showed that customers welcome this innovative way of interacting with them in the retail environment as posited by Davenport et al. (2020) and others, which should give confidence to practitioners considering adoption of AI (Bughin et al., 2017). However, we found that customers’ experiences with online personalisation create very high expectations of the extent of personalisation possible via AI-EP, addressing the gaps identified in Table 1. Those high expectations may be difficult to meet, given not only the technological restrictions of AI-EP but also consumers’ discomfort with location tracking as well as the safeguarding of data which is essential for the efficacy of the offer, which can create customer backlashes and reputation damage (Castillo et al., 2020). Customers’ online experiences also shape their desire for additional services and functionalities, such as the creation of wish lists or the ability to edit their preferences. This desire presents unique challenges from the point of view of interface design which have not been reported, yet. We represented the range of factors impacting positively vs negatively on customers’ experiences with – and assessment of – AI-EP via the motif of Snakes and Ladders boardgame.

Second, we provided empirical evidence of how the impact of the context of message delivery, the format of message delivery and the salience of privacy concerns differs for AI-EP vs online personalisation. Specifically, regarding the impact of the different motivations for online vs. in-store retail on customers’ perception and evaluation of personalisation efforts (Haridasan & Fernando, 2018), we found that customers may be in a particular physical location for reasons other than shopping, and that this may result in heightened irritation from app notifications. Moreover, customers seem more sensitive to evidence of tracking of past purchase behaviour in the physical environment than online, and more likely to resist the tracking of location and shelf-browsing behaviour than online browsing. In terms of the impact of message delivery interface, our findings confirm that the small screen of mobile phones impact negatively on consumers’ involvement with the message (Grewal et al., 2016), and that there is a need for attention-grabbing subject lines to make shoppers want to check the message, immediately. Future research could test the effectiveness of the same message delivered online vs via AI-EP, to quantify the effect of delivery interface on the effectiveness of personalisation campaigns. Another factor that could limit the impact of AI-EP was the high number of notifications that mobile phone users typically receive on their devices, not just from direct messages from other users, but also from social media apps, calendar apps and others. Having said that, AI-EP could be more effective than e-mail offers, possibly because of the relative novelty of this form of personalisation, but also because of the volume of traffic that e-mail may attract (including spam content). Finally, regarding the impact of privacy concerns on consumers’ evaluation of AI-EP, our findings – like Ameen et al (2022)’s study of consumer interactions with smart technologies in shopping malls – seem to contradict Li et al. (2017). Unlike studies of personalisation in the online environment (e.g., Pappas, 2018), customers do not seem too concerned with the firm’s access to their personal information, in principle. This could be because the collection of such information is now seen as a condition for accessing services in the digital era. However, it could also be because of the particular type of app used in our case study. Like Ameen et al (2022)’s app, ours was valid for a shopping area, rather than a specific retailer. This fact may decrease the customers’ perception of surveillance, and increase their trust in the firm behind the AI-EP. Further research is needed to separate the effect of type of app (i.e., retailer vs location specific) from the overall privacy concerns with AI-EP. However, customers did express concerns over access to information which they did not deem essential for the task at hand, and access by unfamiliar retailers. Our findings thus assist in contextualising extant literature on AI-enabled personalisation online vs in-store.

Third, we identified the specific content and process gratifications derived from AI-EP, and how they enhance or detract from the value of AI-EP for retail customers. Content gratifications included discounts, time savings and relevance of offers, with the first one seemingly dominating the others. Receiving notifications on the phone was a process gratification for some but detracted from the overall benefit for others. Likewise, opinions were divided on the process gratification derived from how this app collected and used information for AI-EP. Our findings, thus, extend Sutanto et al (2013)’s work on the manifestation of the personalisation-privacy paradox among smartphone users, in hybrid (physical-digital) environments.

### Practical Contributions

Collectively, these findings mean that the use of AI technology for personalisation in the physical environment can address some of the business challenges faced by high-street retailers as suggested in Davenport et al. (2020), but with significant differences vis a vis personalisation in the online environment. Specifically, our findings have the following managerial implications.

First, AI-EP is more suitable for customer retention efforts, than for customer acquisition. This is both because of the type of dataset required to deliver on customer expectations of AI-EP and avoid the risk of customer backlash, and because of customers’ intense negative reaction to receiving personalised offers from brands that they usually do not buy from. A better way to acquire customers in this demographic group might be through the use of dynamic, entertaining adverts on social media; or by including their items in clothing subscription services (YouGov, 2020).

Second, to attract customers, retailers should offer enticing discounts on desired items. This is because, contrary to the online environment and to what is suggested in the literature (e.g., Kietzmann et al., 2018), we found that customers weren’t driven by hedonic offers, and that there was limited scope for shopping basket expansion.

Third, retailers should focus on providing information about items’ features, availability and other attributes that are important in the pre-purchase stage. This is because, while shoppers may interact with their smartphones across all stages of the purchase process (e.g., Syam & Sharma, 2018), they seemed most receptive to AI-EP offers in the lead-up to the purchase, rather than during the purchase (e.g., payment options) or afterwards (e.g., asking for feedback).

Fourth, retailers need to test various aspects of offer delivery, in order to minimise the concerns and irritants detected in our study. These include the number of notifications, to address shoppers' concerns with battery depletion and the fact that customers may be in the store’s neighbourhood for different reasons; and the wording of the message, to assuage customers’ desire to understand why they got a specific offer. It is also important for retailers to unpack which personalised offers are rejected because customers want to reaffirm their autonomy vs the AI (André et al., 2018), rather than because the offer itself was not persuasive.

Fifth, retailers need to approach data collection and use, carefully. Our study revealed that the use of location and social media data, which is accepted in the online context, caused intense negative reactions among some customers. Conversely, the relative novelty of in-store AI-EP means that customers may be willing to participate in ad-hoc data collection initiatives, if they perceive a link between the information requested and improvements in their shopping experience.

### Research Limitations and Further Research

It is important to recognise the limitations resulting from the focus and characteristics of our approach. The focus on fashion retail, on a multi-store app, and on the UK may limit the transferability of our findings to other research contexts. Research into other empirical settings is needed before claims can be made about consumer perceptions and experiences of AI-EP, generally. Likewise, young female consumers exhibit distinct attitudes to fashion shopping, sharing digital data and interacting with AI, meaning that our findings may not be directly applicable to older female shoppers, or to male shoppers of similar age. Findings from personalisation in the online environment indicate that perception of personalisation benefits is a key a factor in acceptance of personalisation (Pappas et al., 2017). Therefore, it is important to identify which messages most clearly communicate the desired content gratification valued by different types of customers and/or different contexts.

Moreover, by adopting a qualitative approach, we were able to identify a range of issues relevant for fashion retail customers. However, we are not able to quantify their absolute or relative importance. Further research employing quantitative approaches, namely natural experiments (e.g., Tag et al, 2021), is needed before claims can be made about the salience of specific gratifications and privacy concerns, or about the magnitude of their impact on consumer acceptance of AI-EP. Likewise, the use of methodologies such as fuzzy-set qualitative comparative analysis (see Pappas, 2018) would enable the identification of how the different factors identified in this study combine to amplify – or not – purchase intention when exposed to AI-EP.

Furthermore, our focus on consumers overlooks the retailers’ perspective of AI-EP, which is a worthy area of further study. In particular, an avenue of further study that would advance our findings, as well as the work of Yoganathan et al. (2021), is to examine the relationship between AI-EP and access to onsite retail staff, homing in on the digital-physical customer experience dynamic. Given the practical nature of such an investigation, and the need for close collaboration with the organisation deploying the AI-EP solution, it would be beneficial to adopt the clinical inquiry approach method (see Schein, 2008). In this methodological approach, academic researchers and practitioners work together to shape the project, with the explicit goal of improving practice. Clinical inquiry is particularly useful for instigating digital innovation from within the organisation, as demonstrated in Vassilakopoulou et al (2022)’s analysis of the potential for creating hybrid human/AI service teams.

## Footnotes

1 The term “personalisation-privacy paradox” is also, sometimes, used to refer to the disparity between users’ privacy protection intentions and their privacy protection behaviours (e.g., Norberg, Horne & Horne 2007).
